# Predictors of systemic complications and prolonged hospitalization in odontogenic infections: a 12-year retrospective analysis of 997 cases

**DOI:** 10.1186/s12903-025-07517-7

**Published:** 2025-12-12

**Authors:** Martin Fischer, Stefan Schultze-Mosgau, Konrad Tolksdorf

**Affiliations:** Department of Oral and Cranio-Maxillofacial-Surgery/Plastic Surgery, Jena University Hospital, Friedrich-Schiller-University Jena, Am Klinikum 1, Jena, 07749 Germany

**Keywords:** Odontogenic infections, Head and neck infections, Systemic complications, Hospitalization, Multivariable analysis, Risk assessment

## Abstract

**Background:**

Odontogenic infections remain a frequent cause of hospitalization and can progress to systemic complications with substantial resource use. Reliable risk stratification is needed to guide early management. Therefore, this study aimed to identify independent risk factors for systemic complications and prolonged hospitalization in odontogenic infections, and to examine trends in case frequency over a 12-year period.

**Methods:**

A retrospective analysis of 997 surgically treated inpatient cases from 2012 to 2023 was conducted. Clinical outcomes of interest were systemic complications and prolonged hospitalization (≥ 10 days). Associations with clinical and demographic variables were evaluated using bivariate and multivariable logistic regressions. Multivariable models were validated by bootstrap resampling and receiver operating characteristic analyses. Case volume trends were analyzed using Poisson and negative binomial regression models.

**Results:**

Annual hospitalization rates for odontogenic infections increased significantly over the study period (IRR 1.043; 95% CI: 1.025–1.063; *p *< 0.001), despite an overall decline in inpatient admissions. Systemic complications occurred in 3.8% of cases and were independently associated with age (aOR: 1.040 per year; 95% CI: 1.019–1.061; *p* < 0.001), COPD (aOR: 3.707; 95% CI: 1.569–8.761; *p* = 0.003), chronic alcohol addiction (aOR: 5.625; 95% CI: 2.270–13.937; *p* < 0.001), and multi-space involvement (aOR: 5.492; 95% CI: 2.379–12.681; *p* < 0.001). Prolonged hospitalization occurred in 6.7% and was independently predicted by multi-space involvement (aOR: 8.381; 95% CI: 4.390–16.000; *p* < 0.001).

**Conclusions:**

Age, COPD, chronic alcohol addiction, and multi-space involvement independently predict systemic complications in odontogenic infections. Multi-space involvement also identifies patients at risk for prolonged hospitalization. The increasing burden of odontogenic infections highlights the need for a clinical risk score to enable early risk stratification, guide triage, and improve interdisciplinary management while reducing treatment costs.

**Supplementary Information:**

The online version contains supplementary material available at 10.1186/s12903-025-07517-7.

## Background

Odontogenic infections were among the leading causes of death in the early 16th century [1], but advances in dental care, antibiotics, and surgical management have greatly reduced their associated morbidity and mortality [2, 3]. Nevertheless, these infections remain the most common cause of severe head and neck infections [3–5]. They often require emergency hospital admission and are frequently associated with increased complication rates, prolonged hospitalization, and, in rare cases, fatal outcomes [6].

Given the potential severity and lethality of odontogenic infections, it is particularly concerning that recent international studies report a growing incidence of severe cases [7–9]. These infections therefore remain a clinically significant condition and impose a substantial financial burden [10].

In most cases, systemic complications result from the failure to recognize early signs of a severe infection, delayed or incorrect diagnosis, delayed treatment initiation, or inadequate therapy [11]. Given these challenges, it is crucial for treating physicians and dentists to identify high-risk patient groups at an early stage and implement tailored therapeutic strategies to reduce the risk of severe disease courses and potentially life-threatening complications. In such severe cases, the mainstays of therapy include targeted antibiotic treatment, sufficient surgical drainage, endodontic or surgical treatment of the causative teeth, and early interdisciplinary management of emerging complications [7, 12].

However, the predisposing factors leading to severe disease courses and systemic complications remain insufficiently understood [13]. Consequently, further research is needed to identify potential risk factors and high-risk patient groups for severe odontogenic infections.

Therefore, this study aimed to analyze epidemiological data from a university hospital’s patient population to identify and assess risk and predisposing factors for severe courses involving systemic complications and extended hospital stays. In addition, the study investigates whether relevant changes in case frequency can be observed over the study period. The findings are intended to support the early detection of prognostically relevant risk constellations and to enhance treatment strategies in the management of odontogenic infections. In light of these aims, the study also intends to re-establish awareness of the clinical significance and potential severity of odontogenic infections, which continue to pose a considerable yet often underestimated burden on healthcare systems.

## Methods

### Study design and patient population

A clinical-retrospective analysis was conducted using a patient cohort extracted from the digital database of the Jena University Hospital, Germany. Included in the study were all cases treated surgically for odontogenic infections between January 1, 2012, and December 31, 2023, at the Department of Oral and Maxillofacial Surgery/Plastic Surgery of the Jena University Hospital.

**Fig. 1:**
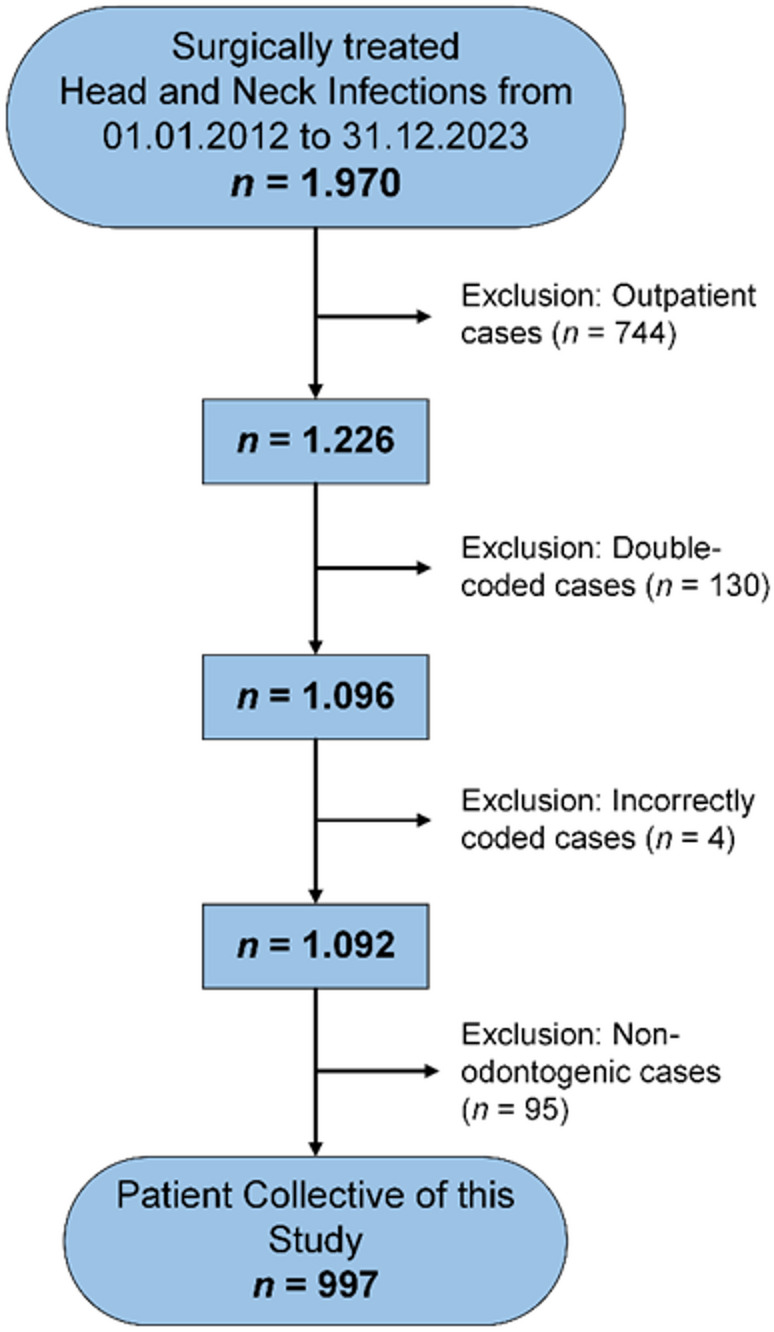
Exclusion criteria of the study population

Case selection was performed using the hospital’s electronic data systems. Identification of odontogenic infection cases was based on International Statistical Classification of Diseases and Related Health Problems, 10th Revision, German Modification (ICD-10-GM) codes (listed in *Supplementary Table 1*), with surgical treatments classified according to the German Operation and Procedure Classification (OPS) codes (listed in *Supplementary Table 2*). Using these criteria, 1,970 inpatient and outpatient cases were identified. Of these, 744 outpatient cases, 130 duplicates, 4 miscoded, and 95 non-odontogenic infections, such as infections secondary to trauma, malignancy, cutaneous infections, non-dentoalveolar procedures, antiresorptive-related osteonecrosis, or osteoradionecrosis, were excluded. The final cohort included 997 inpatient cases of surgically treated odontogenic infections. A detailed flowchart of exclusion criteria is presented in Fig. 1.

### Treatment

Surgical therapy consisted of extraoral and/or intraoral incision and drainage. Where feasible, the odontogenic focus was eliminated in the same procedure.

Standard empirical antibiotic therapy was administered and adjusted based on susceptibility testing when necessary. Ampicillin/Sulbactam or Amoxicillin/Clavulanic acid were administered three times daily, dosed according to body weight and renal function. In patients with reported penicillin allergy, Clindamycin was used.

### Data collection

Data were collected from patient charts. Each case received an anonymized identifier. The following patient-related risk factors and clinical parameters were subjected to further analysis using bivariate and multivariable logistic regression models: age, gender, body mass index (BMI), cardiovascular disease, diabetes mellitus, chronic obstructive pulmonary disease (COPD), obstructive sleep apnea (OSA), severe renal impairment, liver disease, immunosuppression, neurological disease, psychiatric disorders, oral anticoagulation, chronic alcohol addiction, chronic tobacco addiction, self-reported penicillin allergy, and infections involving multiple maxillofacial spaces. Severe renal impairment was characterized as a glomerular filtration rate below 30 mL/min at the time of hospital admission. Immunosuppression was defined as a condition resulting from hematologic diseases, HIV infection, or medical treatment related to organ transplantation, autoimmune disorders, or malignant diseases. Assessment of infection spread to maxillofacial spaces was based on clinical and anatomical documentation, complemented by computed tomography in selected cases.

Due to the low incidence of systemic complications and the relatively high number of potential predictors, most independent variables were dichotomized prior to multivariable analysis to reduce model complexity and enhance the robustness and interpretability of the results. Sociodemographic and anthropometric data were based on the date of initial surgery. Age and BMI were recorded in metric units. Gender, defined as a binary variable based on sex assigned at birth, was categorized as male or female. Risk factors and comorbidities were dichotomized (present / not present). Odontogenic infections were classified as multi-space if ≥ 2 maxillofacial spaces were involved and also dichotomized. No specific maxillofacial space was assigned to local infections.

Inpatient stay was calculated based on the total number of inpatient treatment days. Discharge was usually performed after clinical resolution of the infection. Surgical removal of the causative teeth generally did not lead to an extension of the hospital stay. Cases in which treatment duration was influenced by non-infectious factors (for example discharge against medical advice, unrelated diagnoses or complications, or fatal outcomes) were excluded. The remaining cases were used to determine the adjusted inpatient stay.

The occurrence of systemic complications and prolonged hospitalization were evaluated as the clinical outcomes of interest. The latter was defined as a length of stay exceeding one standard deviation above the mean adjusted treatment duration. In the regression models, both systemic complications and prolonged hospitalization were coded as binary variables.

### Statistical and graphical analysis

Statistical analysis was performed using IBM SPSS Statistics 29.0 (IBM Corporation, Armonk, NY, USA). Significance was set at *p <* 0.05 with 95% confidence intervals (CI). Missing data were excluded from the statistical analyses and indicated in figures and tables by the corresponding reduced sample size (*n*).

Descriptive analyses included means, standard deviations (SD), and ranges (minimum–maximum) for continuous variables, and absolute and relative frequencies for categorical variables.

Patient-related risk factors and clinical parameters were initially analyzed for associations with systemic complications and prolonged hospitalization using bivariate binary logistic regression. Results are reported as odds ratios (OR) with 95% CIs.

Variables with *p <* 0.1 were entered into multivariable logistic regression to account for potential interactions. To prevent overfitting and improve stability, forward likelihood ratio selection was applied when the number of predictors exceeded one tenth of the event rate. Stability and internal validity were further assessed using bootstrap resampling with the bias-corrected and accelerated method based on 5,000 iterations in the context of a low events-per-variable ratio. Variables whose 95% bootstrap CIs included zero were considered non-significant. Multicollinearity was checked via Pearson correlation coefficients (|r| < 0.7). Adjusted odds ratios (aOR) and 95% CIs were reported.

For multivariable logistic regression models, overall model significance was evaluated using the likelihood ratio test (*p <* 0.05). Model fit was assessed using the Hosmer-Lemeshow test with a *p*˗value > 0.05. Both criteria were used as prerequisites for including significant models in the results and are therefore not reported separately. Model performance was further evaluated using Nagelkerke *R²* and the area under the receiver operating characteristic curve (AUC).

To analyze time trends, Poisson regression models were utilized to evaluate the frequency of countable events. Overdispersion was assessed by calculating the ratio of the deviance to the degrees of freedom. If this ratio exceeded 1, a negative binomial regression model was additionally fitted. Final model selection was based on the Akaike Information Criterion and the likelihood ratio chi-square test. Incidence rate ratios (IRR) and their corresponding 95% CIs were reported.

All graphical representations were generated using GraphPad Prism 9 (GraphPad Software, San Diego, CA, USA). Results from multivariable logistic regressions were presented as forest plots showing aORs and 95% CIs. Poisson and negative binomial regressions were illustrated with line charts displaying predicted means and their 95% CIs.

## Results

### Basic characteristics of the study population

Between January 1, 2012, and December 31, 2023, a total of 997 patients were hospitalized and surgically treated for odontogenic infections. Among them, 564 patients (56.6%) were males and 433 (43.4%) females. The average age was 47.8 ± 21.9 years, ranging from 2 to 96 years. The mean hospitalization was 6.2 ± 5.1 days, with a range of 0 to 70 days. Shortened or extended hospital durations were recorded in 80 cases (8.0%) due to discharge against medical advice, in 34 cases (3.4%) due to diagnostics or therapy for unrelated conditions or complications, and in 3 cases (0.3%) due to fatal outcomes. The adjusted mean inpatient stay was 6.0 ± 3.8 days (range: 1–70 days).

An overview of relevant comorbidities and patient-related risk factors is provided in *Supplementary Table 3*. Multi-space involvement occurred in 63 cases (6.3%). Single maxillofacial space involvement was found in 627 cases (62.9%), two spaces in 46 (4.6%), three in 11 (1.1%), and four or more in 6 cases (0.6%). In 307 cases (30.8%), the infections were localized without maxillofacial space involvement. No cases of necrotizing soft tissue infection were observed. The most frequently affected space was the submandibular (50.7%), followed by the buccal (18.6%) and canine fossa (12.0%). An overview of the maxillofacial spaces involved is provided in *Supplementary Table 4*.

Systemic complications occurred in 38 cases (3.8%). An overview of the observed systemic complications is provided in Table 1. Among the 38 patients with systemic complications, 28 (73.7%) required ICU care and 2 (5.3%) died. A total of 67 patients (6.7%) experienced prolonged hospitalization. Intensive Care Unit (ICU) treatment was required in 30 cases (3.0%), predominantly due to systemic complications. Six patients required surgical tracheotomy to secure the airway. In total, three patients (0.3%) died.

**Table 1:** Distribution of systemic complications resulting from odontogenic infections. This table presents the systemic complications that occurred in hospitalized patients with odontogenic infections during the observation period from 2012 to 2023. Absolute case numbers and percentage distributions are reported in relation to the total number of patients included in the analysis (*n* = 997). Several complications could occur simultaneously in the same patient

Complication	Number of cases	Percent (%)
Airway obstruction	21	2,0
Sepsis	19	1,9
Pneumonia	10	1,0
Septic shock	8	0,8
Mediastinitis	5	0,5
Critical illness polyneuropathy	4	0,4
Disseminated intravascular coagulopathy	2	0,2
Brain abscess	1	0,1
Diabetic ketoacidosis	1	0,1
Endocarditis	1	0,1

### Case volume trends

**Fig. 2:**
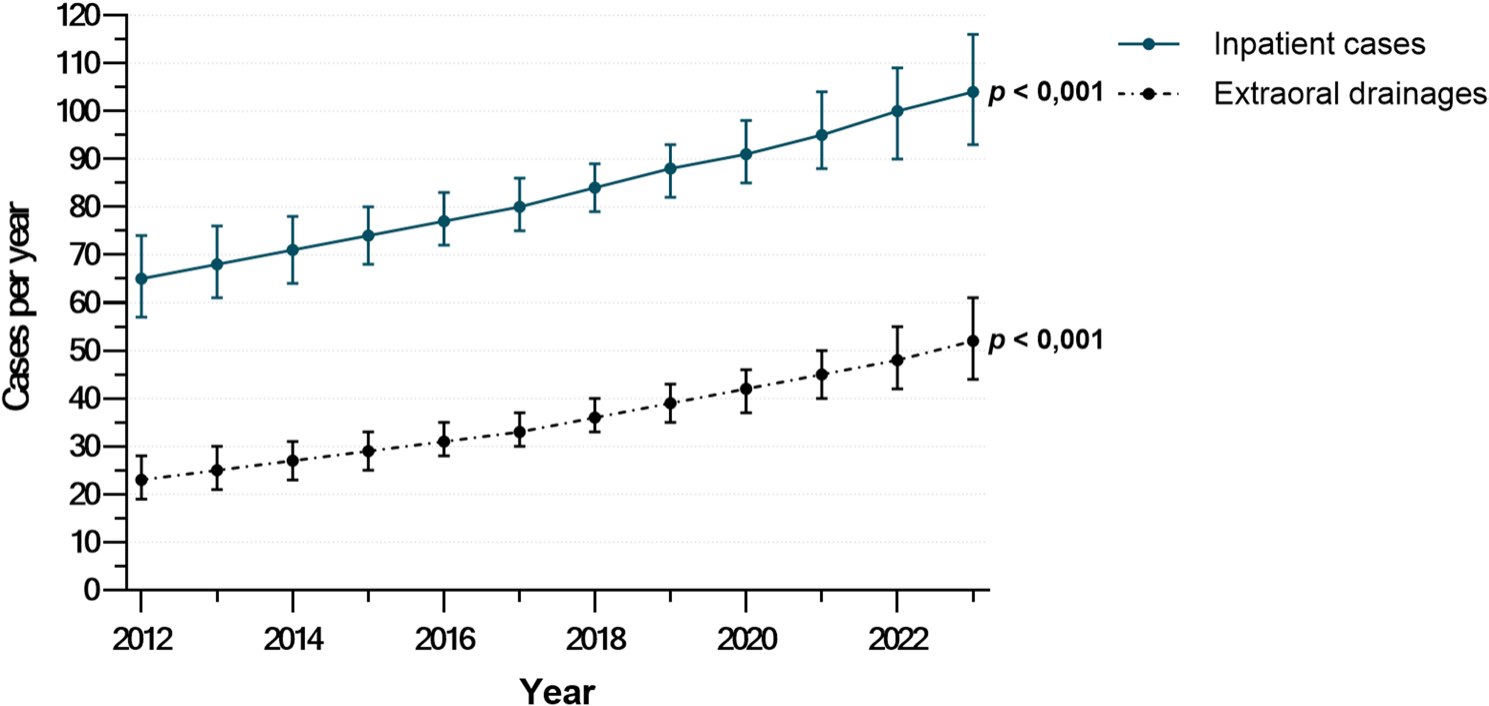
Inpatient cases and extraoral drainages for odontogenic infections from 2012 to 2023. Displayed are the annual case numbers of inpatient treatments and extraoral drainages for surgically treated odontogenic infections during the observation period. The illustrated trends were calculated using Poisson regression models (*n* = 997)

Case volume trends of surgically managed odontogenic infections were analyzed using Poisson regression, with negative binomial adjustment for overdispersion. From 2012 to 2023, inpatient cases increased by 4.3% annually (IRR: 1.043 per year; 95% CI: 1.025–1.063; *p <* 0.001). Extraoral drainage cases rose by 7.5% per year (IRR: 1.075 per year; 95% CI: 1.045–1.106; *p <* 0.001). A graphical overview of inpatient cases and extraoral drainages is shown in Fig. 2.

**Fig. 3:**
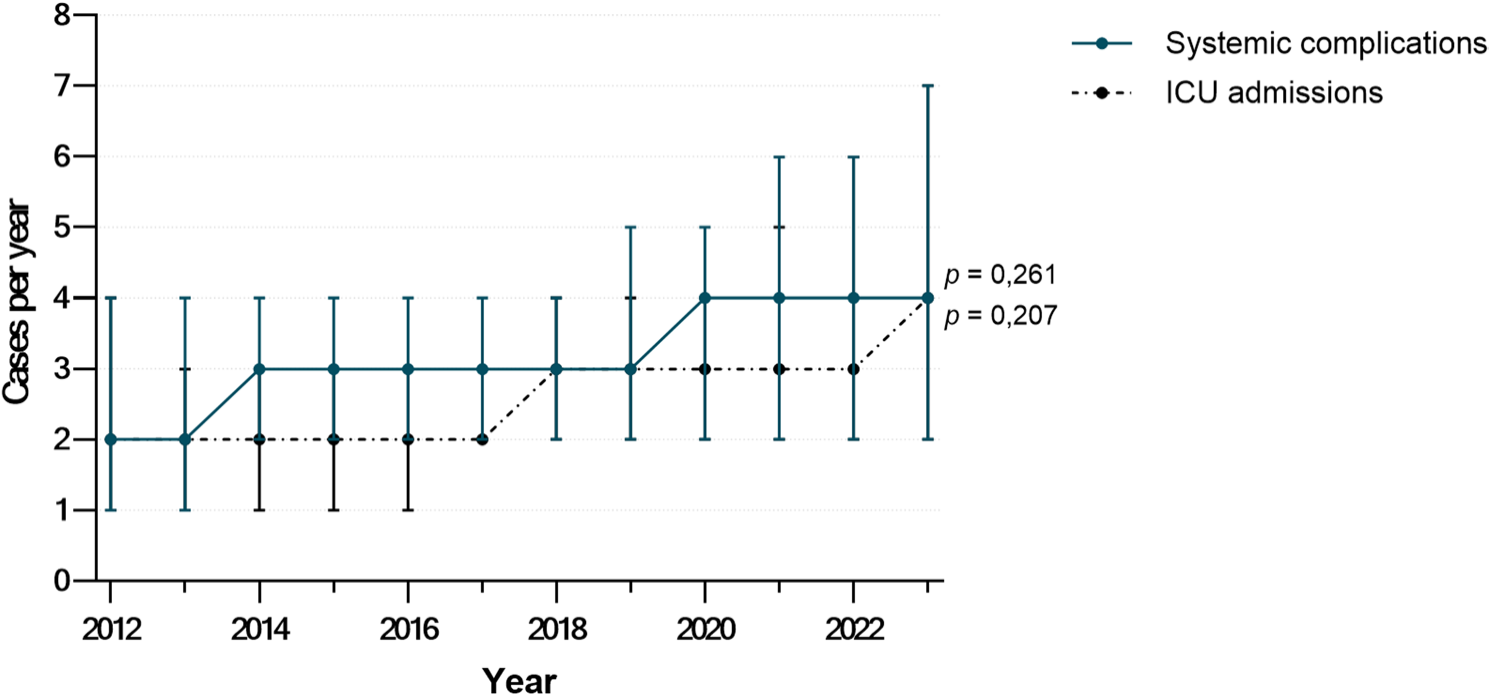
Systemic complications and ICU admissions in odontogenic infections from 2012 to 2023. Displayed are the annual case numbers of systemic complications and ICU admissions in surgically treated odontogenic infections during the observation period. The illustrated trends were calculated using Poisson regression models (*n* = 997)

The incidence of systemic complications increased by 5.5% per year (IRR: 1.055 per year; 95% CI: 0.961–1.158; *p =* 0.261), and ICU admissions by 7.0% per year (IRR: 1.070; 95% CI: 0.963–1.189; *p =* 0.207), though neither trend reached statistical significance. Related trends are shown in Fig. 3.

A comprehensive analysis including all hospital admissions revealed an annual decrease in total inpatient cases by 2.0% (IRR: 0.980; 95% CI: 0.967–0.993; *p =* 0.002). Using total admissions as offset, the share of odontogenic infection cases rose significantly by 6.7% annually (IRR: 1.067; 95% CI: 1.048–1.087; *p <* 0.001). Extraoral drainage cases increased by 10.1% (IRR: 1.101; 95% CI: 1.070–1.133; *p <* 0.001). Systemic complications and ICU admissions increased by 7.9% (IRR: 1.079; 95% CI: 0.982–1.187; *p =* 0.115) and 9.6% (IRR: 1.096; 95% CI: 0.984–1.220; *p =* 0.095), respectively, though not significantly.

### Risk factors for systemic complications and prolonged hospitalization

Bivariate binary logistic regression was used to assess the influence of patient-related risk factors and clinical parameters on the occurrence of systemic complications in odontogenic infections. The results of the bivariate analysis are summarized in Table 2.

**Table 2:** Bivariate logistic regression results of risk factors for systemic complications in odontogenic infections. Displayed are the odds ratios (OR) with corresponding 95% confidence intervals (95% CI) and *p*-values for the occurrence of systemic complications in surgically treated patients with odontogenic infections, depending on the presence of specific risk factors (*n* = 997, unless otherwise specified). Statistically significant results are highlighted in bold

Risk factor	OR	95% CI	*p*-value
Age	1.042	1.024–1.061	**< 0.001**
Gender	0.946	0.493–1.817	0.868
BMI ^a^	1.036	0.991–1.084	0.120
Cardiovascular disease	4.581	2.145–9.784	**< 0.001**
Diabetes mellitus	1.862	0.863–4.019	0.113
COPD	7.798	3.555–17.104	**< 0.001**
OSA	2.133	0.270–16.840	0.472
Severe renal impairment	5.394	1.493–19.492	**0.010**
Liver disease	0.000	0.000 – 0.000	0.999
Immunosuppression	1.924	0.728–5.084	0.187
Neurological disease	2.170	1.003–4.695	**0.049**
Psychiatric disorders	0.909	0.349–2.369	0.845
Oral anticoagulation	4.358	2.251–8.439	**< 0.001**
Chronic alcohol addiction	4.748	2.072–10.881	**< 0.001**
Chronic tobacco addiction ^b^	0.814	0.406–1.634	0.563
Penicillin allergy	1.089	0.327–3.628	0.890
Multi-space involvement	6.105	2.817–13.230	**< 0.001**

^a^*n =* 943 (available BMI data), ^b^*n =* 882 (available tobacco addiction data)

**Fig. 4:**
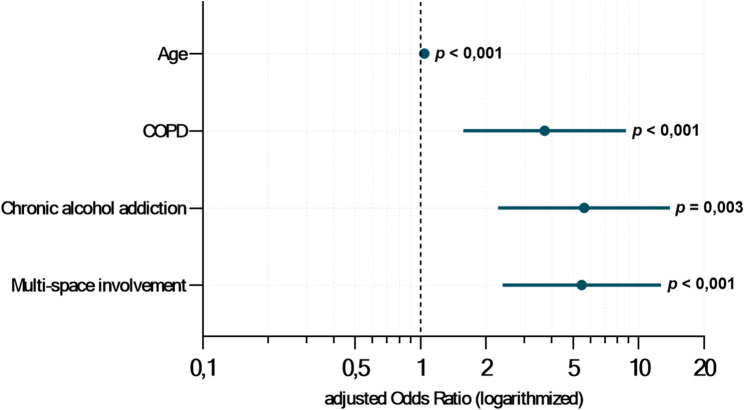
Forest plot of logistic regression showing independent risk factors for systemic complications in odontogenic infections. The forest plot displays the adjusted odds ratios with 95% confidence intervals and *p*-values for the occurrence of systemic complications in surgically treated patients with odontogenic infections, based on the risk factors included in the final regression model (*n* = 997)

Based on the results of the bivariate analysis, the variables age, cardiovascular disease, COPD, severe renal impairment, neurological disease, oral anticoagulation, chronic alcohol addiction, and multi-space involvement were included in further analyses. Forward likelihood ratio selection was applied to identify independent predictors of systemic complications while avoiding model overfitting due to the low event rate. The likelihood ratio test indicated that age (*χ*²(1): 15.892, *p* < 0.001), COPD (*χ*²(1): 19.699, *p* < 0.001), chronic alcohol addiction (*χ*²(1): 9.497, *p* < 0.001), and multi-space involvement (*χ*²(1): 14.356, *p* < 0.001) significantly improved the model and were retained in the final version. The final − 2 log-likelihood was 263.394, compared to 322.837 in the null model. Multivariable logistic regression confirmed that age (aOR: 1.040 per year; 95% CI: 1.019–1.061; *p <* 0.001), COPD (aOR: 3.707; 95% CI: 1.569–8.761; *p =* 0.003), chronic alcohol addiction (aOR: 5.625; 95% CI: 2.270–13.937; *p <* 0.001), and multi-space involvement (aOR: 5.492; 95% CI: 2.379–12.681; *p <* 0.001) were independent predictors of systemic complications. The robustness of the regression estimates was validated through bootstrap analysis (*Supplementary Table 5*). The model showed a Nagelkerke *R*² of 0.209, indicating that approximately 20.9% of the variation in the outcome was explained by the included predictors, and demonstrated good discrimination with an AUC of 0.809. Figure 4 visualizes the multivariable regression results for systemic complications.

Results of the bivariate binary logistic analysis for prolonged hospitalization are shown in Table 3.

**Table 3:** Bivariate logistic regression results of risk factors for prolonged hospitalization in odontogenic infections. Displayed are the odds ratios (OR) with corresponding 95% confidence intervals (95% CI) and p-values for prolonged hospitalization in surgically treated patients with odontogenic infections, depending on the presence of specific risk factors (*n* = 880, unless otherwise specified). Statistically significant results are highlighted in bold

Risk factor	OR	95% CI	*p*-Value
Age	1.023	1.010–1.035	**< 0.001**
Gender	1.026	0.621–1.696	0.921
BMI ^a^	1.020	0.982–1.058	0.309
Cardiovascular disease	2.455	1.462–4.123	**< 0.001**
Diabetes mellitus	1.848	1.020–3.349	**0.043**
COPD	2.315	0.993–5.397	0.052
OSA	3.096	0.644–14.882	0.158
Severe renal impairment	4.238	1.328–13.523	**0.015**
Liver disease	0.000	0.000–0.000	0.999
Immunosuppression	1.673	0.729–3.84	0.225
Neurological disease	0.914	0.425–1.967	0.819
Psychiatric disorders	0.715	0.319–1.604	0.416
Oral anticoagulation	2.502	1.506–4.155	**< 0.001**
Chronic alcohol addiction	1.806	0.738–4.416	0.195
Chronic tobacco addiction ^b^	0.924	0.545–1.568	0.771
Penicillin allergy	1.798	0.819–3.947	0.143
Multi-space involvement	7.649	4.119–14.206	**< 0.001**

^a^*n =* 836 (available BMI data), ^b^*n =* 777 (available tobacco addiction data)

**Fig. 5:**
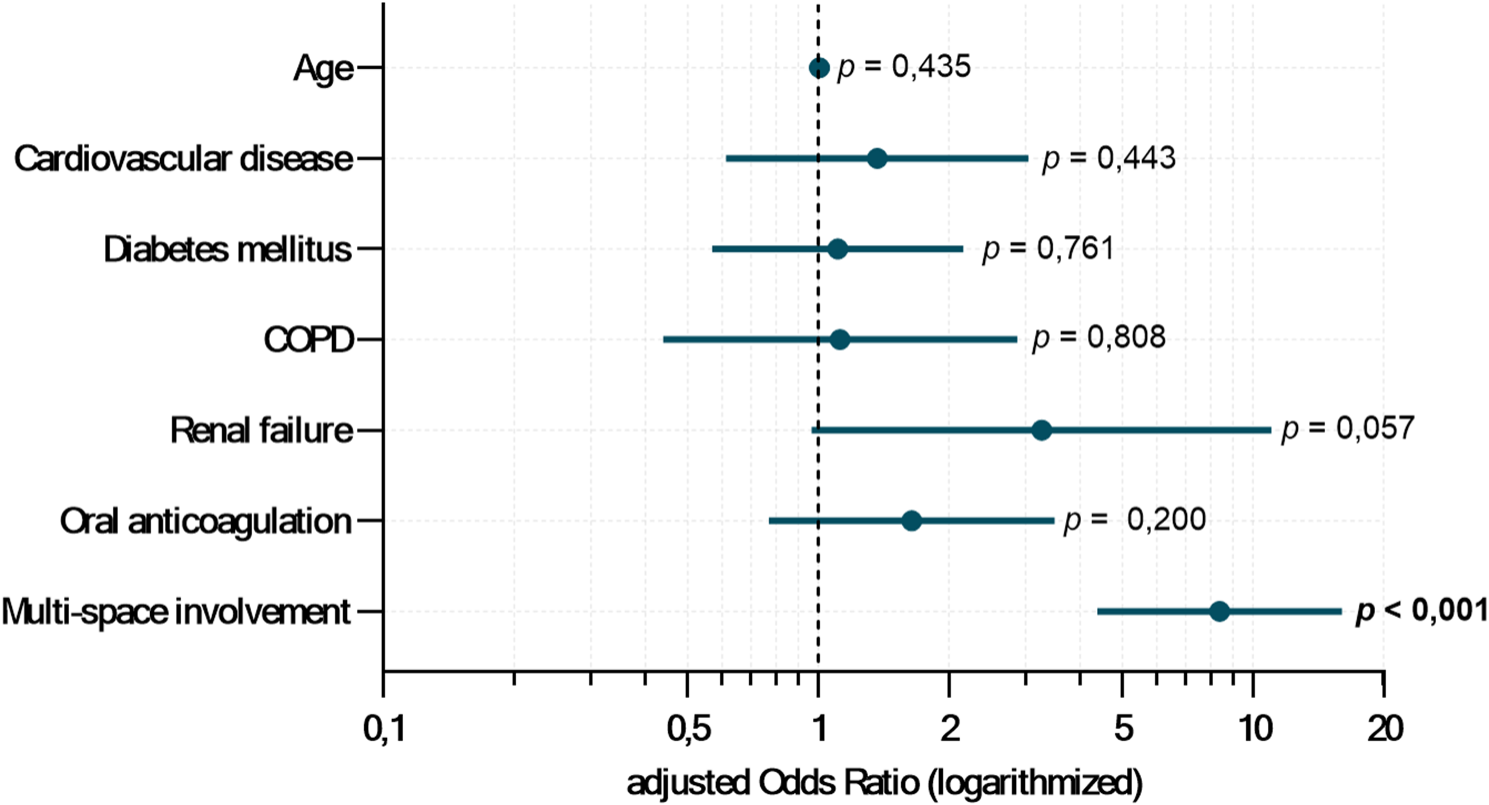
Forest plot of logistic regression showing independent risk factors for prolonged hospitalization in odontogenic infections. The forest plot displays the adjusted odds ratios with 95% confidence intervals and p-values for prolonged hospitalization in surgically treated patients with odontogenic infections, based on the risk factors included in the final regression model (*n* = 880)

Although bivariate analysis showed significant associations for age, cardiovascular disease, diabetes mellitus, severe renal impairment, oral anticoagulation, and multi-space involvement, multivariable analysis identified only multi-space involvement (aOR: 8.381; 95% CI: 4.390–16.000; *p <* 0.001) as a significant independent predictor of prolonged hospitalization. Bootstrap-derived 95% CIs confirmed the stability of this estimate (*Supplementary Table 6*). The model showed a Nagelkerke *R*² of 0.143, explaining approximately 14.3% of the outcome variation, with an AUC of 0.714. Figure 5 illustrates the multivariable regression results for prolonged hospitalization as a forest plot.

## Discussion

Despite the increasing clinical and health-economic burden of odontogenic infections, systematic investigations into risk factors affecting disease progression remain limited. In this context, the present study contributes to a more differentiated assessment of prognostically relevant risk factors and their independent associations with clinical outcomes.

The collected data largely reflects current evidence regarding the incidence and nature of systemic complications in odontogenic infections, as reported rates range between 5% and 27% [13–15]. As indicated in this study, airway obstruction is frequently cited as one of the most common and life-threatening complications, occurring in 40.8% to 75% of systemic cases [15–17], and is considered particularly critical due to its high mortality risk [12, 18, 19]. This highlights the importance of early recognition of airway compromise in clinical practice and the timely initiation of airway management.

This study observed a significant increase in hospitalizations due to odontogenic infections, consistent with current literature [6, 7, 9]. Poisson regression revealed an annual increase in cases, despite a general decline in total inpatient admissions. Additionally, extraoral incisions and drainages increased annually. Although these findings were derived from a single institution, they align with population-based data reported by Meisgeier et al., who observed a significant rise in severe head and neck infections in Germany between 2005 and 2022 (IRR: 1.028; 95% CI: 1.027–1.030; *p* < 0.001) [7]. Notably, the rise observed in our data cannot be explained by population growth, as Thuringia’s population decreased by 3.1% from 2.19 million in 2012 to 2.12 million in 2023 [20], nor by overall hospital admission rates. This suggests that the increasing number of hospitalizations may reflect deficiencies in primary prevention and preclinical management of odontogenic infections [10, 21]. Contributing factors may include an aging population with more comorbidities and rising prevalence of obesity, diabetes, and psychiatric disorders [22–25], as well as changing antimicrobial resistance of the causative bacteria [26]. Socioeconomic disparities also appear influential, as lower income and education levels correlate with higher caries prevalence and tooth loss [27] and reduced dental service utilization [28, 29]. International studies have similarly linked rising head and neck infection incidence to socioeconomically disadvantaged populations [6, 30].

Although Fu et al. and Seppänen et al. reported increased ICU admissions in Australia and Finland [10, 31], this was not statistically confirmed here. However, differences in healthcare systems and ICU admission criteria may limit comparability.

In the multivariable logistic regression model, age (aOR: 1.040 per year), COPD (aOR: 3.707), chronic alcohol addiction (aOR: 5.625), and multi-space involvement (aOR: 5.492) were identified as independent predictors of systemic complications. The model demonstrated good discriminatory capacity (AUC = 0.809), and its explanatory power (Nagelkerke *R²* = 0.209) was within the expected range for clinical models involving multifactorial etiologies [32, 33].

These results not only highlight key risk factors but also reflect patterns observed in previous research. Staffieri et al. and Lee et al. similarly found that multi-space involvement significantly increased the risk of systemic complications [34, 35], while Zhang et al. and Li et al. identified older age as a key factor [15, 19]. However, earlier studies often relied on smaller population sizes and, in some cases, focused primarily on bivariate analyses, which may limit the generalizability and comparability of their findings. In contrast to previous research [17, 36], diabetes mellitus was not independently associated with systemic complications in this cohort (*p =* 0.113). A plausible explanation may be earlier diagnosis and tighter medical surveillance in patients with known comorbidities such as diabetes or immunosuppression, as proposed by Furuholm et al. [13].

The second outcome assessed was prolonged hospitalization, defined as a length of stay at least one standard deviation above the mean. Unlike previous studies that used the mean as a cutoff for prolonged hospitalization [14, 37, 38], this definition more accurately reflects clinical reality by identifying patients with notably extended treatment durations due to increased disease severity or other clinical determinants. As reported hospitalization times for odontogenic infections vary widely across countries, from 2.2 days in Brazil to over 12 days in South Korea [37–40], this relative approach also allows for better international comparability.

In the multivariable logistic regression model for prolonged hospitalization, only multi-space infection (aOR: 8.381; 95% CI: 4.390–16.000; *p <* 0.001) was identified as a significant independent predictor. The final model showed modest explanatory power (Nagelkerke *R*²: 0.143) and moderate predictive ability (AUC: 0.714). Therefore, multi-space involvement emerged as a key determinant of prolonged hospitalization, reinforcing prior evidence of its major impact on disease severity and clinical course [37, 41].

However, certain limitations must be acknowledged. A key limitation of this study is its retrospective design, as data collection relied exclusively on existing clinical records. This may have introduced bias due to incomplete documentation, inconsistent diagnostic criteria, and potential variability in treatment procedures. Dichotomous coding of variables like diabetes and substance use limited insight into clinically relevant gradations, such as severity or dosage effects. It should also be noted that only surgically treated inpatient cases were included. Milder cases treated conservatively or in an outpatient setting were excluded, limiting the generalizability of the results to the broader population of patients with odontogenic infections. Heterogeneity in definitions, inclusion criteria, and treatment protocols across studies further impedes direct comparisons and underscores the need for a standardized classification system in future research.

Despite these constraints, this study highlights key predictors that are consistently associated with an increased risk of systemic complications and prolonged hospitalizations in odontogenic infections. The findings also underscore the importance of multivariable analyses for accurately assessing interrelated risk factors and distinguishing independent predictors. These insights point to the importance of implementing structured pre-hospital risk assessments and interdisciplinary treatment strategies as potential means to prevent complications and reduce hospitalization. Future studies should adopt a prospective, multicenter design, incorporating sociodemographic factors such as educational level and socioeconomic status, as well as microbiological data on detected pathogens and antimicrobial resistance to allow a more detailed assessment of their prognostic relevance. Additionally, research should examine the impact of early, structured care for high-risk patients on complication rates and treatment outcomes. The development of a clinical risk score for odontogenic infections could further support targeted healthcare delivery and improve resource allocation.

## Conclusions

In conclusion, this study identified a significant increase in hospital admissions related to odontogenic infections within the study period, underscoring their clinical and health-economic relevance. In addition, multivariable analysis revealed that age, chronic alcohol addiction, COPD, and multi-space involvement are significant independent predictors of systemic complications in odontogenic infections. Multi-space involvement proved to be a key predictor of prolonged hospitalization, underscoring its relevance for clinical assessment. These findings emphasize the importance of early risk stratification and timely, intensive clinical management to improve outcomes in affected patients. The development of a clinical risk score for assessing the likelihood of complications in odontogenic infections would be highly desirable, as it could enable early identification of high-risk patients and support timely clinical decision-making. Such an approach could improve patient outcomes and promote a more efficient use of healthcare resources.

## Supplementary Information

Supplementary Material 1.

## Data Availability

The datasets used and/or analysed during the current study are available from the corresponding author on reasonable request.

## Abbreviations

aOR: Adjusted odds ratio
AUC: Area under the receiver operating characteristic curve
BMI: Body mass index
CI: Confidence interval
COPD: Chronic obstructive pulmonary disease
ICD-10-GM: International Statistical Classification of Diseases and Related Health Problems, 10th Revision, German Modification
IRRs: Incidence rate ratios
OPS: Germany’s Operation and Procedure Classification
OR: Odds ratio
OSA: Obstructive sleep apnea
