# Supplementary material for: Predictors of systemic complications and prolonged hospitalization in odontogenic infections: a 12-year retrospective analysis of 997 cases
Source: BMC Oral Health. 2025 Dec 12;26:111. doi: 10.1186/s12903-025-07517-7 (PMC12817696; doi:10.1186/s12903-025-07517-7)
Supplement: Supplementary file 1 — Supplementary Material 1. [file 12903_2025_7517_MOESM1_ESM.docx]

**SUPPLEMENTARY FIGURES AND TABLES**

***Supplementary Table 1*: ICD-10-GM codes used for case selection**

| ICD-10-GM code | Description |
| --- | --- |
| H05.0 | Acute inflammation of orbit |
| J34.0 | Abscess, furuncle and carbuncle of nose |
| J36 | Peritonsillar abscess |
| J39.0 | Retropharyngeal and parapharyngeal abscess |
| J39.1 | Other abscess of pharynx |
| K00.7 | Teething syndrome |
| K04.0 – K04.9 | Diseases of pulp and periapical tissues |
| K10.20 – K10.29 | Inflammatory conditions of jaws |
| K10.3 | Alveolitis of jaws |
| K11.3 | Abscess of salivary gland |
| K12.20 – K12.29 | Cellulitis and abscess of mouth |
| L02.0 | Cutaneous abscess, furuncle and carbuncle of face |

***Supplementary Table 2*: OPS** **codes used for case selection**

| OPS code | Description |
| --- | --- |
| **5-240** | **Operations on gums, alveolae and jaw: Incision into the gum and osteotomy of the alveolar ridge** |
| 5-240.0 | Incision into the gum |
| 5-240.1 | Periodontal drainage |
| **5-270** | **Other operations on mouth and face: External incision and drainage in mouth, jaw and face area** |
| 5-270.0 | Temporal |
| 5-270.1 | Periorbital |
| 5-270.2 | Paranasal |
| 5-270.3 | Cheek area |
| 5-270.4 | Parotid region |
| 5-270.5 | Submandibular |
| 5-270.6 | Submandibular, mandibular angle area |
| 5-270.7 | Submental |
| 5-270.8 | Tongue base |
| 5-270.9 | Sternocleidomastoid muscle area |
| 5-270.x | Other |
| 5-270.y | Unspecified |
| **5-273** | **Other operations on mouth and face: Incision, excision and destruction in the oral cavity** |
| 5-273.0 | Incision and drainage, vestibular submucosal |
| 5-273.1 | Incision and drainage, vestibular subperiosteal |
| 5-273.2 | Incision and drainage, sublingual |
| **5-280** | **Operations in the nasopharyngeal and oropharyngeal area: Transoral incision and drainage of a pharyngeal or parapharyngeal abscess** |
| 5-280.0 | (Peri)tonsillar |
| 5-280.1 | Parapharyngeal |
| 5-280.2 | Retropharyngeal |
| 5-280.3 | In the floor of mouth area |
| 5-280.x | Other |
| 5-280.y | Unspecified |

***Supplementary Table 3*: Distribution of comorbidities and patient-related risk factors**

This table presents the relevant comorbidities and patient-related risk factors that occurred in hospitalized patients with odontogenic infections during the observation period from 2012 to 2023. Absolute case numbers and percentage distributions are reported in relation to the total number of patients included in the analysis (*n* = 997, unless otherwise specified). Several comorbidities and patient-related risk factors could occur simultaneously in the same patient.

| Risk factor | Number of cases | Percent (%) |
| --- | --- | --- |
| Cardiovascular disease | 425 | 42.6 |
| Diabetes mellitus | 146 | 14.6 |
| COPD | 52 | 5.2 |
| OSAS | 13 | 1.3 |
| Renal failure | 18 | 1.8 |
| Liver disease | 16 | 1.6 |
| Immunosuppression | 75 | 7.5 |
| Neurological disease | 2 | 0,3 |
| Psychiatric disorders | 142 | 14.2 |
| Oral anticoagulation | 252 | 25.3 |
| Chronic alcohol addiction | 59 | 5.9 |
| Chronic tobacco addiction ^b^ | 406 | 46.0 |
| Penicillin allergy | 73 | 7.3 |
| Multi-space involvement | 63 | 6.3 |

^b^ *n =* 882 (available tobacco addiction data)

***Supplementary Table 4*: Distribution of maxillofacial spaces involved**

This table presents the maxillofacial spaces involved in hospitalized patients with odontogenic infections during the observation period from 2012 to 2023. Absolute case numbers and percentage distributions are reported in relation to the total number of patients included in the analysis (*n* = 783). Several maxillofacial spaces could be affected simultaneously in the same patient.

| Maxillofacial Space | Number of spaces involved | Percent (%) |
| --- | --- | --- |
| Submandibular | 397 | 50.7 |
| Buccal | 145 | 18.6 |
| Canine fossa | 94 | 12.0 |
| Mental/Submental | 41 | 5.3 |
| Para-/Retropharyngeal | 27 | 3.4 |
| Masseterikomandibular | 26 | 3.3 |
| Sublingual | 25 | 3.2 |
| Pterygomandibular | 9 | 1.1 |
| Retromaxillary | 9 | 1.1 |
| Temporal | 5 | 0.6 |
| Parotid space | 2 | 0.3 |
| Orbital | 2 | 0.3 |
| Para-/Peritonsilliar | 1 | 0.1 |

***Supplementary Table 5*: Multivariate logistic regression results of risk factors for systemic complications in odontogenic infections**

Displayed are the regression coefficients (*β*), the adjusted odds ratios (aOR) with corresponding 95% confidence intervals (95% CI), the p-values, and the bootstrap-based 95% confidence intervals (Bootstrap 95% CI) for the occurrence of systemic complications in surgically treated patients with odontogenic infections, based on the risk factors included in the final model (n = 997). Model quality is indicated by Nagelkerke’s *R²* and the AUC. Statistically significant results are highlighted in bold.

| Risk factor | Coeffizient 𝛽 | aOR | 95% CI | *p*-Value | Bootstrap 95% CI |
| --- | --- | --- | --- | --- | --- |
| Age | 0.039 | 1.040 | 1.019 – 1.061 | **< 0.001** | 0.017–0.066 |
| COPD | 1.310 | 3.707 | 1.569 – 8.761 | **0.003** | 0.254 – 2.158 |
| Chronic alcohol,addiction | 1.727 | 5.625 | 2.270 – 13.937 | **< 0.001** | 0.563 –2.707 |
| Multi-space involvement | 1.703 | 5.492 | 2.379 – 12.681 | **< 0.001** | 0.753 – 2.537 |

**Nagelkerke *R^2^***: 0.209**; AUC**: 0.809

***Supplementary Table 6*: Multivariate logistic regression results of risk factors for prolonged hospitalization in odontogenic infections**

Displayed are the regression coefficients (*β*), the adjusted odds ratios (aOR) with corresponding 95% confidence intervals (95% CI), the p-values, and the bootstrap-based 95% confidence intervals (Bootstrap 95% CI) for prolonged hospitalization in surgically treated patients with odontogenic infections, based on the risk factors included in the final model (n = 880). Model quality is indicated by Nagelkerke’s *R²* and the AUC. Statistically significant results are highlighted in bold.

| Risk factor | Coeffizient 𝛽 | aOR | 95% CI | *p-*Value | Bootstrap 95% CI |
| --- | --- | --- | --- | --- | --- |
| Age | 0.007 | 1.007 | 0.989 – 1.025 | 0.435 | -0.012 – 0.026 |
| Cardiovascular disease | 0.314 | 1.368 | 0.614 – 3.048 | 0.443 | -0.561 – 1.117 |
| Diabetes mellitus | 0.103 | 1.108 | 0.570 – 2.155 | 0.761 | -0.601 – 0.715 |
| COPD | 0.116 | 1.123 | 0.440 – 2.868 | 0.808 | -0.959 – 0.923 |
| Renal failure | 1.184 | 3.266 | 0.967 – 11.032 | 0.057 | -0.764 – 1.140 |
| Oral anticoagulation | 0.495 | 1.641 | 0.769 – 3.501 | 0.200 | -0.450 – 2.294 |
| Multi-space involvement | 2.126 | 8.381 | 4.390 – 16.000 | **< 0.001** | 1.428 – 2.823 |

**Nagelkerke *R^2^***: 0.143**; AUC**: 0.714
